# Effects of high-dose dexamethasone on postoperative opioid consumption and perioperative glycaemia in fast-track primary hip arthroplasty: a retrospective cohort study

**DOI:** 10.1007/s00264-025-06430-6

**Published:** 2025-04-03

**Authors:** Rosario Josefina Fabian-Quillama, Tomás Cuñat, Yocelin Saavedra, Elisabet Ripoll-Romero, Nuria Martin, Jenaro Ángel Fernández-Valencia, Montserrat Tió

**Affiliations:** 1 Hospital Clínic of Barcelona, Barcelona, Spain; 2 Departament de Cirurgia i Especialitats Medicoquirúrgiques. Facultat de Medicina i Ciències de la Salut, Universitat de Barcelona, Barcelona, Spain; 3 Departament d’Infermeria de Salut Pública, Salut Mental i Maternoinfantil. Facultat d’Infermeria, Universitat de Barcelona, Barcelona, Spain

**Keywords:** Fast-track, ERAS, Total hip arthroplasty, Dexamethasone, Opioid consumption, Hyperglycaemia

## Abstract

**Background and purpose:**

Standard recommendations for fast-track hip arthroplasty suggest using 8–10 mg of dexamethasone to reduce opioid consumption, with potential benefits of higher doses but scarce data on glycaemic control and complications. This study compares the effects of higher doses versus the standard doses on postoperative opioid consumption, and secondarily, numerical pain scale, glycaemic control, hospital length of stay and postoperative complications.

**Methods:**

Retrospective cohort study of patients scheduled for FAST-TRACK primary hip arthroplasty between 2016 and 2021. Propensity score-matched analyses compared the standard dose group (4–8 mg) versus the high-dose group (16–24 mg).

**Results:**

168 patients were included (56 with 4–8 mg, 112 with 16–24 mg). After one-to-one propensity score matching, 52 patients were included in the standard group and 52 in the high-dose group. After matching, the median [IQR] opioid consumption in the low-dose group was 10 [0–12] and in the high-dose group was 0 [0–10], with a 95% CI of -1 to 0 (*p* = 0.016). In the matched group, there was a median difference of 8 mg/dL (95% CI, -2 to 7, *P* < 0.05) in the immediate postoperative glycaemia, of 17 mg/dl (95% CI, -2 to 14, *P* < 0.05) in glycaemia at 24 h and of -1 day (95% CI, -1 to 0, *P* < 0.05) in hospital stay. No differences in the numerical pain scale and postoperative complications were found.

**Conclusion:**

High-dose dexamethasone slightly increased perioperative glycaemia while reducing opioid consumption and shortening hospital length of stay.

## Background and purpose

Postoperative opioid consumption increases nausea, vomiting, constipation, drowsiness, delirium, orthostatic hypotension, hospital stay and chronic use of opioids [1, 2]. These complications are even more important in hip arthroplasty patients, who tend to be older and frail [2], with 24–62% of them still using opioids after 1 year of surgery [1, 3] and in whom opioid abuse increases mortality (OR 3.7) and morbidity (OR 2.3) [4].

Inadequate control of acute postoperative pain increases persistent postoperative pain and long-term opioid use, all of which can hinder mobility and rehabilitation after hip arthroplasty, increase the risk of venous thromboembolism and prolonged hospital stay [1]. Current perioperative guidelines recommend using multimodal analgesia to improve pain control and reduce postoperative opioid consumption, including dexamethasone for its anti-inflammatory effects [5, 6].

ERAS and PROSPECT recommend 8-10 mg of dexamethasone in fast-track hip arthroplasty [5, 6]. Intravenous dexamethasone is the most common type of administration, but oral dexamethasone has also shown benefits in other types of surgeries such as knee arthroplasty [7]. Single doses, multiple doses and different concentrations have been studied in hip surgery, still with controversy about the optimal dose [7–9].

Standard doses for hip surgery are 4–8 mg, although higher doses (> 8 mg) show potential additional benefits [10–12] but with a theoretical increased risk in morbidity, mortality, infection and suture dehiscence due to hyperglycemia > 180 mg/dL [14, 15]. Nevertheless, there is limited data about the effects of high-dose dexamethasone on perioperative glycemia and complications in hip arthroplasty.

This study aims to compare the effects of higher doses (16–24 mg) versus the standard doses (4–8 mg) on postoperative opioid consumption, and secondarily, numerical pain scale, glycaemic control, hospital length of stay and postoperative complications in patients scheduled for Fast-track primary hip arthroplasty. Additionally, we describe their effect on nausea and vomiting, night rest and time before walking.

## Materials and methods

A retrospective cohort study was carried out and compared two groups: one with low doses of corticosteroids (4–8 mg) and another with high doses (16–24 mg). This study followed the ‘Strengthening the Reporting of Observational Studies in Epidemiology (STROBE)’ guidelines for observational cohort studies.

### Study population

Inclusion criteria were age older than 18 years and patients scheduled for FAST-TRACK primary hip arthroplasty at the Hospital Clinic of Barcelona between 2016 and 2021. Exclusion criteria were a history of chronic pain treatment with strong opioids (fentanyl, methadone, buprenorphine), use of intrathecal morphine during surgery, missing data about opioids or dexamethasone doses and chronic use of corticoids. Baseline clinical and surgical data, preoperative dexamethasone and opioid doses were extracted from medical records and intraoperative anaesthesia charts. Patients were categorized into two groups: standard-dose group ( = < 8 mg) and high-dose group (> 8 mg).

### Outcome measures

All opioid doses were converted to oral Morphine Equivalent Dose (MED). The primary outcome was postoperative opioid consumption defined as the median cumulative oral morphine consumption over 24 h in each group.

Secondary outcomes included median differences between groups on the numerical rating pain scale (NRS, no pain = 0, worst pain possible = 10) at 12 and 24 h after surgery, immediate postoperative glycaemia and 24 h after surgery, hospital length of stay and percentages of postoperative complications (medical, infectious, wound dehiscence or readmission after discharge). Additionally, we described the percentages of nausea and vomiting, adequate night rest and patients with time before walking less than 6 h.

### Sample size and statistical analysis

All analyses were performed using the R statistical software package (V.4.0.2). Based on previous data, we identified that a 30 mg reduction [16] in cumulative 24-hour oral morphine intake was clinically relevant. To detect this difference with a standard deviation (SD) of 12,9 mg [17], we calculated that approximately 15 subjects per group would be needed to reach a statistical power of 80% and a significance level of 0.05, using a two-sample t-test.

A propensity score matching analysis was performed between the standard and high-dose groups to reduce the effect of confounding variables. A logistic regression model was used to estimate the propensity scores of the variables associated with postoperative pain: age, gender, diabetes, ASA physical status, surgical approach, type of anaesthesia, BMI and preoperative VAS score. Using these scores, patients from the standard dose group were matched with the high dose group patients.

A one-to-one propensity score matching without replacement using nearest neighbour matching was performed using the propensity scores. A match was made when a patient in the standard dose group had an estimated propensity score within a calliper width of 0.1 standard deviation of the propensity score of a patient in the high dose group.

Absolute standardised differences were calculated to evaluate the balance of the confounding variables. An absolute standardised difference greater than 0,1 was considered imbalanced. Mann-Whitney test was used for non-parametric numerical outcomes and Chi-squared test was used for categorical outcomes.

### FAST-TRACK protocol

Spinal anaesthesia was preferred over general anaesthesia. All patients received standard intra-operative monitoring and premedication with 1–2 mg midazolam. A consultant anesthesiologist administered spinal anaesthesia and the procedure was conducted in the sitting position with a 25- to 27-gauge needle (BD spinal needle, Quincke point, Becton Dickinson, San Augustín de Guadalix, Spain) at L3–L5 using a standard midline approach (or paramedian if midline is not possible). A total of 10–12.5 mg isobaric (plain) bupivacaine 0.5% was administered depending on the preference of the responsible anesthesiologist. All patients received oxygen through nasal prongs at 2–3 L/min and light sedation with a propofol infusion at 25–50 µg/kg/h. Per institutional protocol, 1 gram of intravenous paracetamol was administered at least 2 h before surgery and an intravenous bolus of tranexamic acid 10 mg/kg was given over 20 min, ending 10 min before the start of the surgery. Tranexamic acid administration continued during the surgery via continuous intravenous infusion at a rate of 2 mg/kg/h until the procedure’s completion.

Dexamethasone was administered after sedation and before surgery, with an initial dose of 4 mg or 8 mg for the first patients in the series. Based on perceived satisfactory results, the dose was gradually increased to 16 or 24 mg at the discretion of the treating team. Since 2019, the protocol recommended a dose of 24 mg for every patient. No periarticular local infiltration anaesthesia was administered.

Patients who received general anaesthesia were induced with lidocaine 1 mg/kg, fentanyl 1.5-2 mg/kg, propofol 1–2 mg/kg and rocuronium 1.2 mg/kg. Maintenance was with intravenous propofol and remifentanyl administered by target-controlled infusion pumps, and a single dose of 3–4 mg of intravenous methadone.

Postoperative pain control involved oral medications, including paracetamol 1 g every 8 h, dexketoprofen 25 mg every 8 h alternately, and morphine 10 mg as rescue doses. Opioids are given when patients report a NRS greater than 3 or when they request a rescue analgesic dose.

## Results

**Fig. 1:**
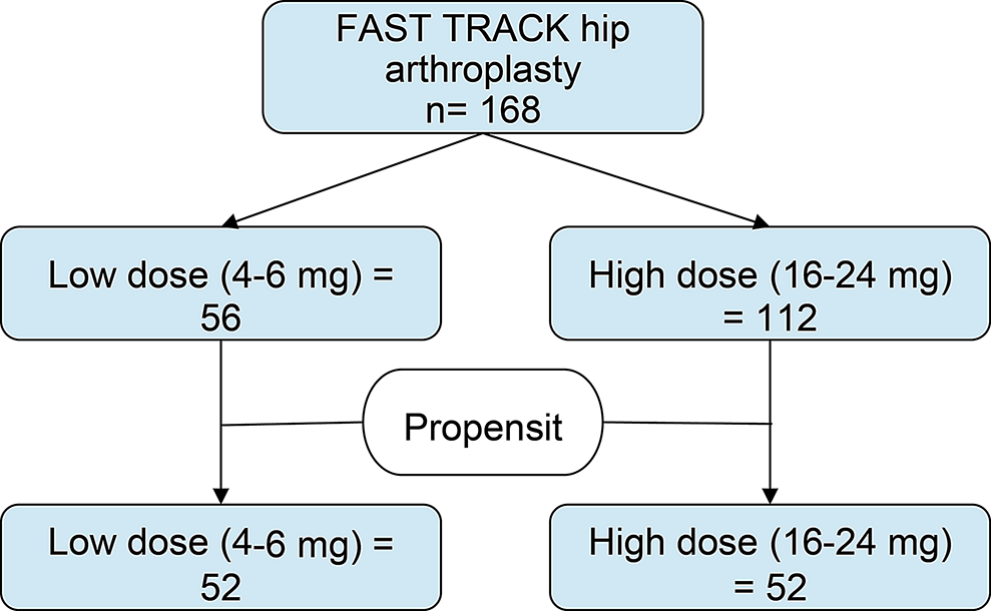
Study flowchart

168 patients met the inclusion and exclusion criteria (Fig. 1). Preoperative dexamethasone was administered at 4, 8, 16 and 24 mg, which were recategorized into low doses ( = < 8 mg, 56 patients) and high doses (> 8 mg, 112 patients).

Baseline clinical characteristics and surgical data before and after propensity score matching are shown in Tables 1 and 2. Before matching, patients’ characteristics were imbalanced in age, gender and surgical approach. After one-to-one propensity score matching, 52 patients were included in the standard group and 52 in the high-dose group. After matching, the pain risk-associated variables were balanced, except for preoperative VAS scores.

**Table 1:** Baseline clinical characteristics and surgical data before matching

	Before propensity score matching		
	Low doses	High doses	SMD
Sample size (168)	56	112	
Age	68.1 (13.1)	65.8 (12.1)	-0.4618
Female	27 (48.2%)	47 (42%)	0.1255
Tobacco	15 (26.8%)	23 (20.5%)	0.14139
DM	10 (17.9%)	16 (14.3%)	0.0458
HTA	21 (60.7%)	60 (42.86%)	-0.2570
BMI	28.1 (4)	27.6 (4.7)	-0.0947
ASA physical status			0.0298
I	4 (7.1%)	15 (13.4%)	
II	41 (73.2%)	67 (59.8%)	
III	11 (19.6%)	29 (25.9%)	
IV	0	1 (0.9)	
Past medical history of PNV	0	1 (0.9)	-0.2448
Apfel scale	1.6 (0.8)	1.4 (0.9)	-0.2280
Preoperative NRS	1.1(1.3)	0.9 (1.3)	-0.1879
Surgical approach			-0.1953
Anterior	2 (3.6%)	30 (26.8%)	
Hardinge	44 (78.6%)	48 (42.9%)	
Posterior	10 (17.9%)	33 (29.5%)	
Type of anesthesia			0.0763
General	9 (16.1%)	15 (13.4%)	
Spinal	47 (87.5%)	97 (86.6%)	
Surgical Time	103.5 (23.6)	106.3 (26.4)	0.1111

Data are mean (SD), median [IQR], and n (%). BMI, Body mass index; SMD, standardised mean difference; Surgical approach, Anterior, Hardinge or Posterior; NRS, numerical rating scale

**Table 2:** Baseline clinical characteristics and surgical data after matching

	After propensity score matching		
	Low doses	High doses	SMD
Sample size (168)	52	52	
Age	67.1 (12.9)	66.6 (12)	0.039
Female	24 (46.2%)	23 (44.2%)	0.0385
Tobacco	15 (28.8%)	10 (19.2%)	0.2626
DM	9 (17.3%)	7 (13.5%)	< 0.001
HTA	30 (57.7%)	24 (46.2%)	-0.215
BMI	27.8 (4)	28.3 (4.7)	0.122
ASA physical status			0.031
I	4 (7.7%)	9 (17.3%)	
II	39 (75%)	29 (55%)	
III	9 (17.3%)	13 (25%)	
IV	0	1 (1.9%)	
Past medical history of PNV	0	0	< 0.001
Apfel scale	1.5 (0.8)	1.4 (1)	-0.1992
Preoperative NRS	1 (1.17)	0.8 (1.1)	-0.1849
Surgical approach			0.03
Anterior	2 (3.9%)	12 (23%)	
Hardinge	41 (78.9%)	20 (38.5%)	
Posterior	9 (17.3%)	20 (38.5%)	
Type of anesthesia			0.055
General	8 (15.4%)	7 (13.5%)	
Spinal	44 (84.6%)	45 (86.5%)	
Surgical Time	102.3 (23.6)	105.3 (24.8)	0.1246

Data are mean (SD), median [IQR], and n (%). BMI, Body mass index; SMD, standardised mean difference; Surgical approach, Anterior, Hardinge or Posterior; NRS, numerical rating scale

Primary and secondary outcomes before and after matching are shown in Tables 3 and 4. After matching, the median [IQR] morphine consumption in the low-dose group was 10 mg and in the high-dose group was cero (Table 4).

**Table 3:** Numerical primary and secondary outcomes before propensity score matching

Before propensity score matching				95% CI		
	Low doses	High doses	Median difference	Lower limit	Upper limit	*P* value
Hospital LOS (days)	2 [2–4]	2 [1–3]	-1	-1	0	< 0.001*
Postoperative NRS at 12 h	1 [1–2]	1 [1–1]	0	0	0	0.98
Postoperative NRS at 24 h	1 [1–2]	1 [0–1]	0	0	0	0.07
NRS post mobilization	2 [1–3]	1 [1–3]	0	-1	0	0.01*
Immediate postoperative glycemia (mg/dL)	89 [80.5-103.8]	97 [89-110.5]	8	-2	7	0.007*
24 h glycemia (mg/dL)	118 [101.8-139.5]	132 [120–154]	17	-2	14	0.002*
Morphine equivalents in the first 24 h (mg)	10 [0–12]	0 [0–10]	0	0	0	0.004*

Data are mean (SD), and median [IQR]. SMD, standardized mean difference; NRS, numerical rating scale; LOS, length of stay. *P* value compares the low doses group and the high doses group. A one-sided paired Mann-Whitney test was used to compare medians between the groups. *Indicates that a statistical significance level < 0.05 was achieved

**Table 4:** Numerical primary and secondary outcomes after propensity score matching

After propensity score matching				95% CI		
	Low doses	High doses	Median difference	Lower limit	Upper limit	*P* value
Hospital LOS (days)	2 [2–4]	1.5 [1–3]	-1	-1	0	0.011*
Postoperative NRS (12 h)	1 [1–2]	1 [0–1]	0	0	0	0.602
Postoperative NRS (24 h)	1 [1–2]	1 [0–1]	0	0	0	0.047*
NRS post mobilization	2 [1–3]	1 [1–3]	0	-1	0	0.26
Immediate postoperative glycemia (mg/dL)	89.5 [82-103.7]	98.5 [90–109]	8	-1	9	0.027*
24 h glycemia (mg/dL)	118 [101.8-139.5]	132 [120–155]	17	0	16	0.003*
Morphine equivalents in the first 24 h (mg)	10 [0–12 ]	0 [0–10]	0	-1	0	0.016*

Data are mean (SD), and median [IQR]. SMD, standardized mean difference; NRS, numerical rating scale; LOS, length of stay. *P* value compares the low doses group and the high doses group. A one-sided paired Mann-Whitney test was used to compare medians between the groups. *Indicates that a statistical significance level < 0.05 was achieved

**Table 5:** Categorical secondary outcomes before matching

	Before propensity score matching			
	Low doses	High doses	*p*-value	SMD
PNV	7 (12.5%)	10 (8.9%)	0.65	0.1181
Adequate night rest	50 (89.3%)	99 (88.4%)	1	0.0281
Time before walking (< 6 h)	40 (71.4%)	73 (65.2%)	0.52	0.1328
Immediate postoperative glycemia above 180 mg/dL	2 (3.6%)	2 (1.8%)	0.47	
24 h glycemia above 180 mg/dL	5 (2.7%)	19 (1.8%)	0.16	
Complications until 90 postoperative days:			0.39	-0.2717
Infectious complication	2 (3.6%)	3 (2.7%)	1	-0.0524
Mechanical complication	4 (7.1%)	5 (4.5%)	0.72	-0.1186
Wound dehiscence	3 (5.4%)	1 (0.9%)	0.21	-0.2920
Readmission after discharge	5 (8.9%)	3 (2.7%)	0.13	0.0890

*P*-value comes from Chi-squared test. N (%); PNV: postoperative nausea and vomits

In the matched group, the median difference in immediate postoperative glycaemia was 8 mg and the median difference in glycaemia at 24 h was 17 mg/dl (Table 4). Median values and their interquartile ranges were less than 180 mg/dL.

There was a median difference of -1 days of hospital stay (95% CI, -1 to 0, *P* < 0.05) for the matched group.

There was no association between standard and high dexamethasone doses with NRS at 12 h, 24 h after surgery or after mobilization (Tables 3 and 4). There was no difference in the percentage of postoperative complications (infectious, mechanical, wound dehiscence and readmission after discharge), postoperative nausea and vomits, wound dehiscence or readmission after discharge), night rest or time before walking (Tables 5 and 6).

**Table 6:** Categorical secondary outcomes after matching

	After propensity score matching			
	Low doses	High doses	*p*-value*	SMD
PNV	7 (13.5%)	7 (13.5%)	1	< 0.001
Adequate night rest	47 (90.4%)	49 (94.2%)	0.71	-0.143
Time before walking (< 6 h)	37 (71.2%)	38 (73.1%)	1	-0.043
Immediate postoperative glycemia above 180 mg/dL	2 (1.9%)	1(1%)	1	
24 h glycemia above 180 mg/dL	5 (4,8%)	10 (9,6%)	0.26	
Complications until 90 postoperative days:			0.66	-0.1465
Infectious complication	2 (3.9%)	0	0.48	-0.2787
Mechanical complication	4 (7.7%)	4 (7.7%)	1	0
Wound dehiscence	2 (3.9%)	1 (1.9%)	1	-0.1143
Readmission after discharge	5 (9.6%)	1 (1.9%)	0.09	0

*P-value comes from Chi-squared test. N (%); PNV: postoperative nausea and vomits

## Discussion

In this study, patients receiving high-dose dexamethasone (16 mg or 24 mg) before surgery showed a statistically significant reduction in cumulative opioid consumption within the first 24 h compared to those receiving lower doses (4–8 mg). Previous studies corroborate these findings. A systematic review indicated that administering more than 10 mg of dexamethasone is required to achieve significant postoperative pain reduction [18], which is consistent with the doses used in this study. Furthermore, a meta-analysis of randomized controlled trials demonstrated that intravenous corticosteroids, including dexamethasone, effectively manage pain and facilitate early rehabilitation in patients undergoing total knee or hip arthroplasty, notably reducing the need for rescue opioids [19]. Nevertheless, despite our observed reduction in opioid consumption, it did not reach the 30 mg threshold of clinical relevance [16]. Moreover, the optimal dosing range and perioperative administration schedule, whether as a single dose or multiple doses, remain to be determined.

In our case, we observed a median difference of -1 day which was statistically significant, however, our study lacks power to state if it is clinically relevant.

Some literature suggests that the cortisol suppression effect may last up to a week. However, a double-blind controlled trial showed that the peak cortisol suppression was at 24 h post dexamethasone [21]. Thus, we considered that 24 h of follow-up is enough to detect cortisol-related complications. In our study, we observed a median difference in immediate postoperative glycaemia of 8 mg/dL and a median difference of 17 mg/dL at 24 h between the low-dose and high-dose corticosteroid groups, with median and interquartile ranges of postoperative glycaemia remaining under 180 mg/dL, which is the threshold for clinical significance [20, 22]. These findings are consistent with previous literature about lower doses, which did not find an association of dexamethasone to the odds of having postoperative glucose levels > 200 mg/dl [23, 24]. However, there was a higher percentage of blood glucose greater than 180 mg/dL in the high-dose matched group (4.8% vs. 9.6%, Table 6), although not statistically significant. Future studies should expand on the follow/up of hyperglycemia and its treatment, which was not possible in this study because of missing data.

All of this suggests that it is safe to use high doses without causing perioperative hyperglycaemia. In other studies the increase in glycaemia was dependent on the past medical history of diabetes, so this group of patients should have stricter follow up and management [22].

Neither complication in the first three months after surgery was associated with high-dose dexamethasone and is coherent with previous data [20]. Interestingly, one study described that using dexamethasone did not increase the odds of periprosthetic joint infection while lowering the odds of other postoperative infections in diabetes patients [15].

Nonetheless, our study was subject to several limitations associated with the retrospective character of the data. First of all, we had a few cases with 8 mg or less of dexamethasone to compare as the current hospital protocol for hip surgery recommends the administration of 24 mg of dexamethasone. For this reason, we collected data from before the implementation of that protocol to have enough patients in the comparison group. However, low-dose patients data was also collected from the period after 2019, as the protocol was a recommendation and some anaesthesiologists preferred lower doses in the first years of the implementation. Of notice, our hospital protocol recommends the use of regional anaesthesia with bupivacaine 0.5% 10–12 mg intrathecal, a single dose of intravenous dexamethasone before surgical incision, sedation with propofol perfusion in TCI, and postoperative pain control with paracetamol 1 g every 8 h, dexketoprofen every 8 h alternate and morphine as rescue doses. We remark on this because a randomised trial showed that the combination of paracetamol, ibuprofen, and dexamethasone had the lowest morphine consumption within 24 h following surgery in comparison to just paracetamol and ibuprofen [25], so future studies about dexamethasone should also include a combination of paracetamol plus a non-steroidal anti-inflammatory drug.

We could not include other risk factors associated with opioid use after total hip arthroplasty such as race, depression or anxiety, history of substance abuse, chronic pulmonary disease, acquired immunodeficiency syndrome, peripheral vascular disease, history of non-specific chronic pain and back pain [3]. These factors should be included in prospective studies. Although it is well known that chronic opioid users have higher and problematic postoperative opioid use [1, 3], in this study we excluded this type of patient to explore only the effect of high-dose dexamethasone in patients with less complexity. For this reason, it would be interesting to develop prospective studies to evaluate if high doses of dexamethasone still have an opioid-sparing effect in patients with long-term opioid use.

Despite using propensity score matching to reduce confounding, the number of variables we were able to balance was limited by the few cases with low doses. We prioritized the variables more related to postoperative pain and the variables that could not be balanced had overlapping confidence intervals. For instance, the difference in the upper limits at CI 99.7% in the preoperative NRS scores were 4.51 and 4.1, with a difference of only 0.41 which is likely not clinically significant, but we cannot rule out its influence on the pain-related outcomes.

The propensity score matching method, while robust, does not entirely eliminate confounding, and residual confounding may still be present. Our sample was also unbalanced for tobacco and the Apfel Scale. This may explain why we couldn’t find an association between the use of high-dose dexamethasone and nausea and vomiting. Regarding tobacco and postoperative pain there is limited evidence linking both of them and we could not find studies about it in hip surgery. Future studies would need to add the amount of cigarettes, which was not possible in our case.

Meta-analyses have demonstrated that patients undergoing direct anterior hip replacement experience reduced pain intensity in the early postoperative days compared to those undergoing the posterior approach [26]. Additionally, the direct anterior approach yields superior clinical outcomes when compared to other surgical approaches [27]. These benefits are supported by lower levels of acute-phase reactants, such as CRP, IL-6, and ESR, as observed in various studies [26]. Conversely, the posterior approach is associated with a higher risk of failure to discharge in the outpatient total hip replacement setting [28]. However, the subject is controversial in literature and one study has shown that neither surgical approach (direct anterior, anterolateral or posterior) is associated with opioid usage over 180 days after surgery [29]. Our study was not powered to draw strong conclusions regarding the surgical approach.

## Conclusions

This study shows that high-dose dexamethasone is associated with a statistically significant although not clinically relevant reduction in opioid use in Fast-track hip arthroplasty and a decrease in hospital stay of 1 day. Future studies including the risk factors not evaluated in this work may show a better approach to the opioid-sparing effects of high-dose dexamethasone. These high doses seem safe as there was a non-clinically relevant increase in perioperative glycemia and no difference in the percentage of complications at three months.

## Data Availability

No datasets were generated or analysed during the current study.
